# Orofacial symptoms and diagnostic pathways in patients with giant cell arteritis (GCA): a retrospective case series from a dental perspective

**DOI:** 10.1186/s12903-026-08669-w

**Published:** 2026-05-21

**Authors:** Tina Willmen, Lukas Willmen, Katja Döring, Meike Stiesch, Annette Doris Wagner

**Affiliations:** 1 Clinic of Prosthetic Dentistry and Biomedical Materials Research, Hannover Medical School, Hanover, Germany; 2 Department of Nephrology and Hypertension, Hannover Medical School, Carl-Neuberg-Straße 1, Hannover, 30625 Germany; 3 Institute for Diagnostic and Interventional Neuroradiology, Hannover Medical School, Hanover, Germany

**Keywords:** Temporomandibular disorders, Giant cell arteritis, Rare diseases, Diagnostic delay, Jaw claudication

## Abstract

**Background:**

Giant cell arteritis (GCA) is a rare systemic vasculitis that primarily affects medium-sized and large arteries and frequently involves the extracranial branches of the carotid artery, including the temporal artery. Orofacial symptoms may occur that clinically resemble temporomandibular disorders (TMD) or other dental conditions and thus represent a particular diagnostic challenge in dental practice. Data on orofacial manifestations and diagnostic pathways in biopsy-confirmed GCA from a dental perspective remain limited. The aim of the present study was to analyze orofacial symptoms, diagnostic pathways, and factors that may contribute to delayed diagnosis.

**Methods:**

This retrospective descriptive single-center case series included six patients with biopsy-confirmed GCA treated at a tertiary outpatient clinic for rare systemic inflammatory diseases. Medical records were systematically reviewed, and missing information was supplemented through patient interviews conducted by telephone or in person using a predefined interview guide. These interviews were used to reconstruct symptom onset, orofacial manifestations, warning signs, and diagnostic pathways. The findings were analyzed descriptively and summarized in tabular and narrative form.

**Results:**

All six patients exhibited orofacial manifestations. Dental consultation formed part of the diagnostic pathway in five cases and represented the first professional point of contact in three. Load-related chewing complaints consistent with jaw claudication were documented in four cases, while atypical orofacial presentations were also observed. Elevated C-reactive protein (CRP) and fatigue/malaise were present in all patients; headache or temporal pain and visual symptoms occurred in five cases each. Time to diagnosis ranged from approximately 4 weeks to 5 months.

**Conclusion:**

Orofacial symptoms may represent a clinically relevant component of GCA presentation, and dental care may be involved early in the diagnostic pathway. In patients older than 50 years, atypical, progressive, or treatment-resistant orofacial symptoms, particularly when accompanied by cranial or systemic warning signs or visual impairment, may indicate a non-dental cause and warrant further medical evaluation.

**Trial registration:**

Not applicable. This retrospective descriptive case series was not registered in a public clinical trial registry.

## Background

Approximately 15% of rare diseases manifest in the oral and maxillofacial region [1]. Giant cell arteritis (GCA) represents one such entity [2]. It predominantly affects individuals over the age of 50, with the majority of cases occurring between 70 and 79 years of age [2, 3]. Females are affected twice as often as males [2]. GCA is a form of vasculitis that primarily affects medium-sized and large arteries, including the arteries of the aortic arch and the extracranial branches of the carotid arteries. Its relevance to dental medicine arises from possible involvement of the external carotid artery and its branches. The maxillary artery, superficial temporal artery, and facial artery supply the masticatory muscles, the temporomandibular joint, and extensive parts of the orofacial region. Inflammatory luminal narrowing of these arteries may result in load-related ischemic symptoms such as jaw claudication. In addition, GCA may present with headache or temporal pain, scalp tenderness, visual symptoms, constitutional complaints, and symptoms associated with polymyalgia rheumatica (PMR) [2, 4, 5]. PMR is a closely associated inflammatory rheumatic condition typically characterized by bilateral shoulder and/or pelvic girdle pain and morning stiffness [6]. The clinical presentation of GCA varies depending on the pattern of vessel involvement. As some of these symptoms overlap with dental complaints or manifestations of temporomandibular disorders (TMD), patients may present for dental evaluation early in the diagnostic course. Shenoy et al. stated that general dental practitioners may encounter approximately five to six patients with GCA over the course of their professional careers [7]. Dentists therefore play a particular role in identifying symptoms that are not primarily dental in origin, facilitating timely medical evaluation, and thereby helping to prevent diagnostic delays.

Diagnostic delay has been described as a relevant challenge in GCA. Prior et al. reported a mean time to diagnosis of nine weeks [5]. In the dental setting, if systemic or visual accompanying symptoms are not reported by patients or specifically elicited during history-taking, orofacial symptoms may initially be plausibly interpreted as dental or TMD-associated complaints. This may delay further medical evaluation and contribute to diagnostic latency. Limited awareness of, and familiarity with, rare diseases such as GCA among dental professionals may further impede timely referral. While dentists’ knowledge of GCA has not been specifically investigated, survey data from Germany and Israel indicate knowledge gaps among dental professionals regarding rare diseases with orofacial manifestations [8, 9].

In cases of GCA, a delayed diagnosis can lead to serious consequences such as irreversible visual loss, aortic aneurysm, aortic dissection, or cerebrovascular events, which may be associated with increased mortality [4, 5].

Given that orofacial symptoms, accompanying warning signs, and the role of early dental presentations in the diagnostic course of biopsy-confirmed GCA have so far been investigated only to a limited extent, the aim of this analysis was to describe orofacial symptoms, systemic and cranial warning signs, and diagnostic pathways in patients with biopsy-confirmed GCA, with particular focus on the role of the initial dental consultation and on features suggestive of a non-dental cause of the symptoms.

## Methods

This study was designed as a retrospective, descriptive, single-center case series and was conducted at the Outpatient Clinic for Rare Systemic Inflammatory Diseases with Renal Involvement at Hannover Medical School (MHH), Hanover, Germany, a university-affiliated tertiary care center. The clinic specializes in the diagnosis and management of rare systemic inflammatory diseases with renal involvement and provides interdisciplinary expertise in nephrology, rheumatology, and rare diseases.

### Case selection

At the time of data collection, the medical records of all living patients with a confirmed diagnosis of GCA who were under follow-up at the outpatient clinic and for whom consent for supplementary data collection could be obtained were systematically screened for eligibility. Inclusion required histopathologically confirmed GCA based on a positive temporal artery biopsy. Additional inclusion criteria were that the diagnostic course could be sufficiently reconstructed from the available records, that orofacial and/or cranial symptoms were documented, and that the confirmed diagnosis had been established at least six months before data collection. Cases with insufficient documentation, a diagnostic pathway that could not be sufficiently reconstructed, or a presentation without orofacial or cranial symptoms were excluded.

### Data collection

For each included case, data collection was based on a structured review of medical records, supplemented by telephone or in-person follow-up inquiries with the patients, guided by an interview guide. The clinical course from symptom onset to confirmed diagnosis was reconstructed from the available documentation, including digital and paper-based records from Hannover Medical School as well as external medical or dental reports from the period preceding diagnosis. Extracted information included symptom onset and symptom characteristics, orofacial complaints, initial dental or medical consultations, accompanying systemic symptoms, laboratory findings, histopathological confirmation by temporal artery biopsy, available diagnostic procedures including vascular ultrasonography, PET/CT, MRI, CT, and scintigraphy where documented, and the temporal sequence and content of the diagnostic work-up before the confirmed diagnosis.

To supplement missing information and clarify incomplete or ambiguous details, all included patients were contacted by telephone or in person. These follow-up inquiries were limited to factual information and complemented the chart review, particularly where the available records did not fully capture the chronological sequence of symptoms, the order of consultations, or clinically relevant details. Where additional medical or dental records were relevant and available for reconstructing the diagnostic course, these were requested after obtaining patient consent.

### Data analysis

Given the small number of cases, analysis was limited to descriptive methods. The findings were summarized in tabular and narrative form, with a focus on recurring clinical patterns, diagnostic pathways, and potential contributors to diagnostic delay.

## Results

### Study population and diagnostic pathway

At the time of data collection, ten living patients with confirmed GCA were receiving follow-up care at the outpatient clinic and were assessed for eligibility. Four patients were excluded: three because their presentation was limited to non-cranial large-vessel involvement without documented cranial or orofacial manifestations and one because the diagnostic pathway could not be reconstructed with sufficient reliability. The final case series comprised six patients with biopsy-confirmed GCA. The cohort included four female and two male patients, with age at symptom onset ranging from 65 to 75 years.

**Fig. 1:**
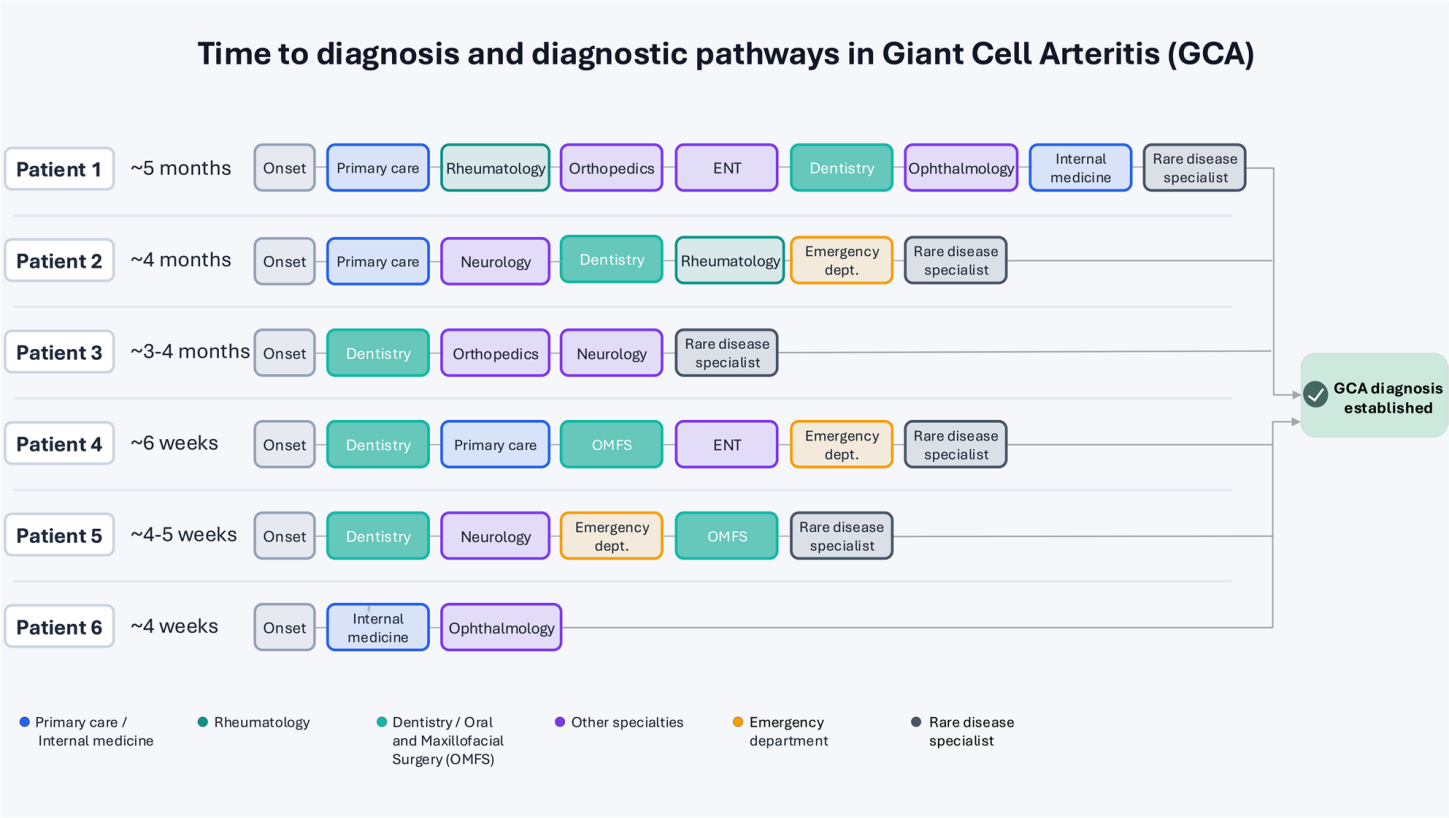
Time to diagnosis and diagnostic pathways in six patients with GCA. The figure shows the chronological sequence of healthcare contacts from symptom onset to confirmed diagnosis and primarily serves to visualize the diagnostic course and the sequence of involved healthcare contacts rather than to provide a complete quantitative count of all individual contacts. For clarity, repeated contacts with the same clinician, department, or institution are not displayed separately. The total number of distinct healthcare settings involved before diagnosis is reported in Table 1. The colors indicate the different healthcare settings involved. The time spans listed on the left show the approximate duration from symptom onset to diagnosis

First presentation occurred in dental care in three cases, in primary care in two cases, and in internal medicine in one case. The number of distinct healthcare settings involved before diagnosis ranged from 3 to more than 10, and emergency presentation was documented in four patients (Table 1; Fig. 1). Temporal artery biopsy confirmed GCA in all six patients. However, the diagnosis was not based on histopathology alone but was interpreted within the overall clinical context, including compatible symptoms, elevated inflammatory markers, histopathological findings, and adjunctive imaging. Vascular ultrasound was performed in all six patients and showed findings compatible with GCA. PET/CT was performed in four patients, and MRI was used in selected cases as part of the extended diagnostic work-up.

**Table 1:** Patient characteristics and diagnostic context

Patient	Sex	Age at onset (y)	First symptom	First health care contact	Distinct health care settings before diagnosis (*n*)	First GCA suspicion	Emergency presentation
1	M	67	Heel complaints; later ear / preauricular pain	Primary care	>10	GP after temporal artery symptoms	✓ (Eye clinic, acute visual loss)
2	F	75	Severe headache	Primary care	6	Rheumatologist	✓
3	M	69	Symmetrical back pain	Dentistry (telephone consultation)	4	Neurologist	-
4	F	70	Bilateral mandibular pain	Dentistry	6	Rare disease specialist	✓
5	F	65	Right mandibular pain	Dentistry	5	Rare disease specialist	✓
6	F	73	Subfebrile temperatures, fatigue, weight loss	Internal medicine	3	Internal medicine	-

This table summarizes demographic characteristics, initial symptoms, first healthcare contact, the number of distinct healthcare settings involved before diagnosis, first suspicion of GCA, and emergency presentation. Repeated contacts with the same clinician, department, or institution were counted once, whereas contacts with different clinicians, departments, or institutions were counted separately

### General symptoms and systemic warning signs

General symptoms and clinical warning signs were frequent in the present study population (Table 2). Fatigue/malaise were documented in all six patients. Weight loss was reported in five patients and ranged from 2 to 6.5 kg, while subfebrile temperatures were noted in two cases. Elevated inflammatory parameters were present in all six patients, with C-reactive protein (CRP) elevated in all cases. Other inflammatory parameters, including erythrocyte sedimentation rate (ESR) and leukocyte count, were elevated in some, but not all, patients. Headache and/or temporal pain occurred in five patients. Visual symptoms were likewise present in five patients and ranged from subjective visual deterioration to diplopia, visual field defects, and transient visual loss or amaurosis-like episodes. Temporal artery tenderness and/or abnormality was documented in five patients, and scalp tenderness in three. PMR or PMR-related symptoms were documented in three patients, including patients with a pre-existing diagnosis of PMR before GCA was diagnosed.

**Table 2:** General symptoms and systemic warning signs

Patient	Headache/temporal pain	Scalp tenderness	Temporal artery tenderness/abnormality	Visual symptoms	Inflammatory markers elevated	Weight loss	Fatigue/malaise	Fever/subfebrile	Polymyalgia rheumatica (PMR)
1	✓	-	✓	✓	✓	2 kg	✓	✓	✓
2	✓	✓	✓	✓	✓	6.5 kg	✓	-	-
3	✓	-	✓	✓	✓	6 kg	✓	-	✓
4	✓	✓	✓	✓	✓	4 kg	✓	-	-
5	-	-	-	-	✓	-	✓	-	✓
6	✓	✓	✓	✓	✓	3-4 kg	✓	✓	-

The table summarizes cranial and systemic warning signs in the six included patients

### Orofacial symptoms and initial dental assessment

Orofacial manifestations were documented in all six cases (Table 3). Symptoms were predominantly bilateral and mainly involved the jaw/ear/preauricular region as well as the temporal region. In four cases, load-related chewing complaints were described, consistent with jaw claudication, including pain on chewing and/or jaw fatigue. Pain on speaking or mouth opening was reported only occasionally, including possible restricted mouth opening in one case and indirect discomfort during speaking in another. Tongue- or taste-related symptoms were uncommon and were reported in only one case in the form of taste impairment.

**Table 3:** Orofacial symptoms and initial dental assessment

Patient	Dentist consulted	Reason for dental consultation	Main pain location	Laterality	Pain character	Pain on chewing	Jaw claudication	Jaw fatigue	Pain on speaking/mouth opening	Tongue/taste symptoms	Initial dental assessment	Dental diagnostics	Dental treatment	Response
1	✓	Jaw/ear/preauricular complaints	Jaw/ear/preauricular region	Bilateral, right >left	Stabbing ear pain, load-related	✓	✓	✓	Limited mouth opening	-	TMD	Panoramic radiograph	Occlusal splint therapy	No relief
2	✓	Rule out dental cause	Temporal/above ear	Bilateral, left > right	Persistent	-	-	-	-	-	Dental cause excluded	Clinical dental assessment	n.a.	n.a.
3	✓	Jaw pain	Jaw/temporal region	Bilateral	Severe, load-related	✓	✓	✓	-	-	Dental cause excluded; musculoskeletal/TMD-related pain	Recent unremarkable dental assessment	n.a.	n.a.
4	✓	Mandibular pain	Mandible + temporal/ face	Bilateral	Load-related	✓	✓	✓	-	Taste impairment	TMD	Panoramic radiograph/OMFS assessment	Occlusal splint therapy	No relief
5	✓	Right mandibular pain	Right mandible	Right	Neuralgiform, intermittent	-	-	-	Discomfort during pain attacks	-	Suspected trigeminal neuralgia	Panoramic radiograph, vitality testing	n.a.	n.a.
6	-	n.a.	Jaw + temporal region	Bilateral, left >right	Load-related pulling/tearing jaw pain	✓	✓	✓	-	-	n.a.	n.a.	n.a.	n.a.

This table summarizes the orofacial symptoms and chewing-related complaints of the six included patients, as well as the initial dental assessment, dental diagnostics, and dental treatment. A dash indicates that the feature was not reported, and “n.a.” indicates “not applicable”

In five of the six cases, dental consultation formed part of the diagnostic course; in three cases, dentistry represented the first healthcare contact relevant to the subsequent diagnostic work-up. This first healthcare contact did not always coincide with the earliest symptoms: in one of these cases, back pain initially remained without medical assessment, and telephone-based dental advice was sought after jaw pain had developed.

In two of the five cases involving dental consultation, a dental cause was excluded at an early stage and onward referral was initiated.

In one additional case, despite the presence of jaw pain, a dental cause was also excluded early without dental consultation because the patient’s medical background prompted immediate evaluation in internal medicine. Overall, dental assessments ranged from exclusion of a dental cause to a musculoskeletal or TMD-related interpretation and suspected trigeminal neuralgia. In two cases, occlusal splint therapy, as a form of oral appliance therapy, was initiated for presumed muscular TMD; however, no symptom improvement was observed, and further worsening of symptoms was described instead. Where referral from the dental setting occurred, patients were referred to oral and maxillofacial surgery, orthopedics, neurology, or otorhinolaryngology.

## Discussion

While GCA is well described in the medical literature, it has so far been addressed only to a limited extent from a dental perspective, despite the potential relevance of its orofacial manifestations to differential diagnosis in dental practice.

In the present case series, all included patients had biopsy-confirmed GCA, which strengthens the diagnostic certainty of the observations. However, temporal artery biopsy does not provide complete diagnostic sensitivity, as vascular inflammation in GCA may be segmental and skip lesions or insufficient biopsy length can contribute to false-negative biopsy findings [10].

In this cohort, orofacial manifestations of the disease were documented in all six patients. Dental consultations formed part of the diagnostic pathway in five of the six cases and represented the first professional point of contact in three. Load-related chewing complaints consistent with jaw claudication were documented in four cases.

Jaw claudication is one of the most clinically informative symptoms of GCA and typically manifests as load-dependent pain or fatigue of the masticatory muscles. Pathophysiologically, jaw claudication in GCA is considered to result from ischemia of the masticatory muscles due to arteritis of branches of the external carotid circulation, particularly the maxillary artery. Vascular inflammation with intimal thickening and luminal narrowing reduces perfusion during functional activity, leading to exertional jaw pain or fatigue during chewing that typically improves when mastication is stopped [11]. This pattern differs from masticatory myofascial pain, which is primarily non-ischemic and is usually characterized by localized muscle tenderness and reproduction of familiar pain during palpation or jaw movement [12]. In contrast to GCA-related jaw claudication, myofascial pain is not typically accompanied by systemic inflammatory signs, visual symptoms, temporal artery abnormalities, or elevated inflammatory markers.

In a systematic review and meta-analysis of 68 studies including 14,037 patients, jaw claudication showed one of the highest positive likelihood ratios among clinical symptoms (LR + 4.90; 95% CI 3.74–6.41) [13]. Larger cohorts also underline the relevance of craniofacial symptoms: Chean et al. reported that 143 of 318 patients had pain or difficulty chewing at the time of diagnosis, and 59 of 318 reported toothache [14]. From a dental perspective, this is particularly relevant because load-related jaw pain, temporal pain, and preauricular discomfort may initially resemble myogenous symptoms in the context of TMD. In addition, vasculitis-related tenderness along the course of the temporal artery may be misinterpreted as tenderness of the temporalis muscle. This diagnostic overlap was also reflected in the present case series: in two cases, occlusal splint therapy was initiated for presumed muscular TMD without symptom relief. These observations are also supported by the limited dental literature on GCA. Published reports on individual patients describe cases presenting with symptoms such as toothache, jaw pain, or pain when chewing [15–18]. In some cases, these symptoms were initially misinterpreted as temporomandibular or myofascial in origin [11, 15, 19, 20]. In a subset of cases, TMD-oriented treatment was initiated [15, 19, 21], and in one case splint therapy was explicitly described [21], before GCA was identified as the underlying condition. Taken together, these reports underline that GCA may initially mimic a dental or TMD-associated symptom pattern.

The diagnostic trajectories observed in the present case series were often complex and involved multiple healthcare settings before the final diagnosis was established. Time to diagnosis ranged from approximately four weeks to five months; in one case, more than ten different healthcare settings were involved. In four cases, the diagnostic course escalated to emergency care. The meta-analysis by Prior et al. also showed that diagnostic delay varies according to clinical presentation, with average delays of 7.7 weeks in cranial GCA and 17.6 weeks in non-cranial GCA [5]. A retrospective fast-track study by van Nieuwland et al. confirmed this difference, reporting a median delay of 21 days in isolated cranial GCA compared with 57 days in patients with extracranial large-vessel involvement [22]. Notably, most of the delay occurred before referral, whereas specialized assessment was usually performed within one day after referral [22]. The complexity observed in the present case series may therefore reflect the overlap of early GCA manifestations with common differential diagnoses encountered in primary care and dental practice. This is clinically relevant, as delayed diagnosis in GCA may be associated with severe complications.

For dental practice, the key issue is less establishing the diagnosis of GCA than recognizing symptom constellations that are not readily explained by a primary dental cause and should prompt timely medical evaluation. GCA typically affects patients older than 50 years and may present with new-onset headache or temporal pain, jaw claudication, scalp tenderness, visual symptoms, constitutional complaints, temporal artery abnormalities, and elevated inflammatory markers [10, 23, 24]. PMR, an inflammatory rheumatic condition, is closely associated with GCA; Nielsen et al. reported a pooled point prevalence of 42% for PMR at the time of GCA diagnosis [23]. In the present series, all patients were older than 60 years; weight loss was reported in five cases, fatigue or malaise in all six, and three patients had PMR or PMR-associated symptoms. Against this background, dentists should be particularly alert when newly developed jaw, temporal, or preauricular complaints are load-dependent, progressive, or refractory to treatment and occur alongside constitutional symptoms, visual complaints, PMR-associated features, or elevated inflammatory markers [25, 26]. In such constellations, prompt medical assessment and referral should be considered [25, 26]. Visual symptoms are especially important, as delayed detection may lead to irreversible ischemic complications, including permanent vision loss [25–27]. Inspection and palpation of the temporal artery may provide additional clues, with tenderness, nodularity, or reduced pulsation being classically described findings; rare manifestations such as tongue pain or tongue claudication may also occur [26].

### Limitations

This analysis is limited by its retrospective design and the small number of included cases (*n* = 6), which is not unexpected given the rarity of the disease. In addition, potential selection bias must be considered, as the cases come from a specialized tertiary outpatient clinic for rare systemic inflammatory diseases. The included patients may therefore be more likely to represent particularly complex or diagnostically challenging cases of GCA. Parts of the diagnostic pathway had to be reconstructed from patient recall, which may have introduced recall bias. However, the interviews focused on factual aspects of the diagnostic process, such as the presence or absence of specific symptoms, consultations, and treatments, rather than on subjective assessments. The interview data were cross-referenced with available medical records whenever possible and generally supported the documented clinical course. The findings are descriptive and should be interpreted cautiously. Because of the small number of cases, they cannot be generalized but may help generate further hypotheses.

## Conclusion

The present analysis suggests that orofacial symptoms may represent a clinically relevant component of GCA presentation and that dental care is often involved early in the diagnostic pathway. In this case series, atypical, progressive, or treatment-resistant symptom constellations, particularly when accompanied by cranial or systemic warning signs, were not always compatible with a primarily dental cause. This underlines the potential role of dentists in the early detection of such symptom patterns and in referring patients for further medical investigations.

## Data Availability

The data supporting the findings of this study are available from the corresponding author (ADW) upon reasonable request. The data are not publicly available because they contain information that could compromise patient privacy.

## Abbreviations

CI: confidence interval
CRP: C-reactive protein
ENT: ear, nose and throat
ESR: erythrocyte sedimentation rate
GCA: giant cell arteritis
GP: general practitioner
LR+: positive likelihood ratio
MHH: Hannover Medical School
MRI: magnetic resonance imaging
OMFS: oral and maxillofacial surgery
PET/CT: positron emission tomography/computed tomography
PMR: polymyalgia rheumatica
TMD: temporomandibular disorders
